# Involvement of TLR2 in Recognition of Acute Gammaherpesvirus-68 Infection

**DOI:** 10.1371/journal.pone.0013742

**Published:** 2010-10-29

**Authors:** François Michaud, François Coulombe, Éric Gaudreault, Jasna Kriz, Jean Gosselin

**Affiliations:** 1 Laboratory of Innate Immunology, Centre Hospitalier Universitaire de Québec Research Center (Centre Hospitalier de l'Université Laval) and Department of Molecular Medicine, Faculty of Medicine, Université Laval, Quebec, Canada; 2 Centre Hospitalier Universitaire de Québec Research Center (Centre Hospitalier de l'Université Laval) and Department of Psychiatry and Neurosciences, Faculty of Medicine, Université Laval, Quebec, Canada; Louisiana State University, United States of America

## Abstract

**Background:**

Toll-like receptors (TLRs) play a crucial role in the activation of innate immunity in response to many viruses. We previously reported the implication of TLR2 in the recognition of Epstein-Barr virus (EBV) by human monocytes. Because murine gammaherpesvirus-68 (MHV-68) is a useful model to study human gammaherpesvirus pathogenesis *in vivo*, we evaluated the importance of mouse TLR2 in the recognition of MHV-68.

**Methodology/Principal Findings:**

In studies using transfected HEK293 cells, MHV-68 lead to the activation of NF-κB reporter through TLR2. In addition, production of interleukin-6 (IL-6) and interferon-α (IFN-α) upon MHV-68 stimulation was reduced in murine embryonic fibroblasts (MEFs) derived from TLR2−/− and MyD88−/− mice as compared to their wild type (WT) counterpart. In transgenic mice expressing a luciferase reporter gene under the control of the mTLR2 promoter, MHV-68 challenge activated TLR2 transcription. Increased expression levels of TLR2 on blood granulocytes (CD115^−^Gr1^+^) and inflammatory monocytes (CD115^+^Gr1^+^), which mobilized to the lungs upon infection with MHV-68, was also confirmed by flow cytometry. Finally, TLR2 or MyD88 deficiency was associated with decreased IL-6 and type 1 IFN production as well as increased viral burden during short-term challenges with MHV-68.

**Conclusions/Significance:**

TLR2 contributes to the production of inflammatory cytokines and type 1 IFN as well as to the control of viral burden during infection with MHV-68. Taken together, our results suggest that the TLR2 pathway has a relevant role in the recognition of this virus and in the subsequent activation of the innate immune response.

## Introduction

MHV-68 is a virus naturally present in rodent populations [1]. It is genetically and biologically related to the two major human gammaherpesviruses, Epstein-Barr virus (EBV) and Kaposi's sarcoma-associated herpesvirus (KSHV) [2]. MHV-68 is known to infect its host through nasopharyngeal tissues where a primary lytic replication occurs in lung epithelial cells. Replication in the lungs subsequently leads to infected cell migration to the lymphoid tissues and establishment of a latent state mainly in B lymphocytes [3], [4], [5], [6] and macrophages [7].

During the last decade, several reports have highlighted the crucial role of the TLR system in host defense against microbial agents. In this regard, many members of the herpesvirus family have already been shown to activate TLRs. For example, different cell populations infected by herpes simplex virus 1 or 2 (HSV-1 and HSV-2) were found to secrete robust levels of type 1 IFN, IL-6 and tumor necrosis factor-α (TNF-α) through the activation of TLR2 and TLR9 [8], [9], [10]. Human cytomegalovirus (CMV) and varicella-zoster virus (VZV) are other members of the herpesvirus family known to activate an inflammatory response through TLR2 [11], [12]. In addition, we previously showed that EBV is recognized by TLR2 and that this recognition event leads to monocyte chemotactic protein-1 (MCP-1) secretion by human monocytes [13]. Regarding MHV-68, recent studies have shown interactions between this virus and the TLR system. For example, the increase of lytic and latent viral loads observed in spleen, but not in lungs of TLR9−/− mice as compared to control groups suggest that TLR9 is important in organ-specific immunity against MHV-68 during both lytic and latent infection [14]. The MyD88 adaptor protein is required for downstream signaling by all TLRs except TLR3. In its absence, a decrease in the frequency of MHV-68 viral genome-positive B cells was observed in spleens of mice suggesting that the MyD88 pathway contributes to the control of MHV-68 latency establishment in B cells [15]. Lastly, the transcription factor NF-κB, which can be activated through MyD88, also seems to regulate MHV-68 latency establishment and its maintenance in B cells [16]. In fact, NF-κB p50−/− mice showed increase and persistent viral replication in lungs when compared to WT mice, suggesting that NF-κB p50 is required for immune control by the host.

While cytokine production during infection of mice with MHV-68 has been reported [14], [17], little is known about the contribution of the TLR system in such production. In addition, a recent report identified TLR2 as a mediator of type 1 IFN secretion in response to viral ligands [18]. Because several herpesviruses were reported to activate TLR2, we investigated the implication of this TLR in the early innate response against MHV-68. Our results indicate that during acute infection with MHV-68, TLR2 signaling via MyD88 is an important aspect of the proinflammatory and antiviral responses directed against this virus.

## Results

### MHV-68 activates NF-κB through TLR2

**Figure 1:**
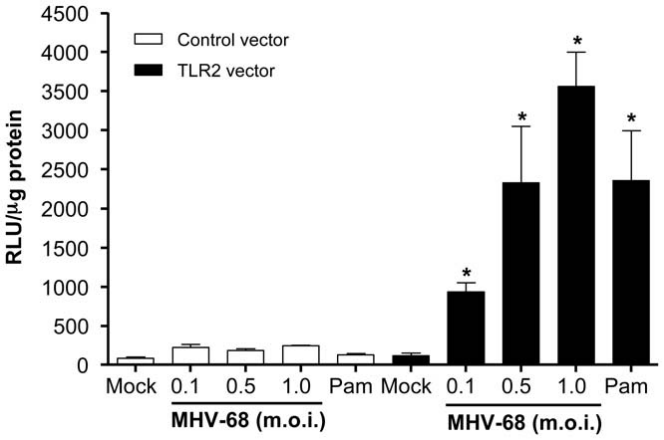
MHV-68 activates TLR2 in transfected HEK293 cells. HEK293 cells were transiently transfected with either murine TLR2 or control plasmid (400 ng) along with NF-κB luciferase reporter plasmid (100 ng). Forty-eight hours post-transfection, cells were stimulated with mock, infectious MHV-68 (m.o.i. 0.1, 0.5 and 1) or Pam_3_CSK_4_ (Pam, 1 µg/ml) for 6 hours followed by luciferase assay. Data are representative of two different experiments. Values that were significantly different (*p*<0.05) from mock control are indicated (*).

We previously demonstrated that EBV induces MCP-1 secretion by human primary monocytes via the TLR2/NF-κB signaling pathway [13]. In order to investigate whether MHV-68 could be recognized by TLR2, HEK293 cells were transiently co-transfected with a control vector or with a murine TLR2 (mTLR2) expression plasmid along with a NF-κB luciferase reporter plasmid. Transfected cells were then stimulated with increasing multiplicities of infection (m.o.i.) of MHV-68 or with the synthetic TLR2 ligand Pam_3_CSK_4_. NF-κB activation, as measured by luciferase activity, was observed in TLR2-expressing cells stimulated with Pam_3_CSK_4_ indicating functional TLR2 expression (Figure 1). Upon MHV-68 stimulation, dose-dependent NF-κB activation was a consequence of TLR2 triggering by the virus since no significant luciferase activity was observed in cells transfected with the control vector. Thus, this first set of experiment shows that MHV-68 has the capacity to induce the activation of NF-κB in transfected cells expressing mTLR2.

### TLR2 and MyD88 are involved in MHV-68-induced production of IL-6 and type 1 IFN

**Figure 2:**
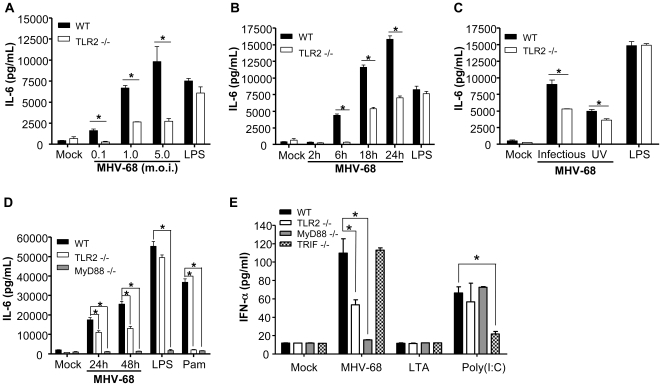
Inflammatory response to MHV-68 in MEFs is dependent on TLR2 expression. (**A**) WT and TLR2−/− MEFs were stimulated with mock, infectious MHV-68 at indicated m.o.i. or LPS (1 µg/ml) for 18 hours. Cell-free supernatants were then assessed for IL-6 secretion by ELISA assay. (**B**) WT and TLR2−/− MEFs were stimulated with mock or infectious MHV-68 (m.o.i. of 1) for indicated periods of time or with LPS (1 µg/ml, 18 hours). Cell-free supernatants were then assessed for IL-6 secretion. (**C**) WT and TLR2−/− MEFs were stimulated with mock, infectious MHV-68, UV-irradiated MHV-68 (m.o.i. of 1) or LPS (1 µg/ml) for 24 hours and cell-free supernatants were then assessed for IL-6 secretion. (**D**) WT, TLR2−/− and MyD88−/− MEFs were stimulated with mock or infectious MHV-68 (m.o.i. of 1) for 24 hours and 48 hours or with LPS (1 µg/ml) or Pam_3_CSK_4_ (Pam, 1 µg/ml) for 48 hours and cell-free supernatants were then assessed for IL-6 secretion. (**E**) WT, TLR2−/−, MyD88−/− and TRIF−/− MEFs were stimulated with mock or with infectious MHV-68 (m.o.i. of 1), LTA (10 µg/ml) or poly(I:C) (5 µg/ml, transfected with Lipofectamine) for 24 hours. Levels of IFN-α were then determined in cell-free supernatants by ELISA. Data are representative of three different experiments. *p<0.05.

TLR2 activation leads to the production of proinflammatory cytokines via the MyD88 adaptor molecule. Since MEFs express all TLRs in a functional state and can secrete a wide range of cytokines [19], we isolated this cell type from WT and various mutant mice in order to study TLR2-dependent cytokine secretion in response to MHV-68. First, WT and TLR2−/− MEFs were stimulated with lipopolysaccharide (LPS) or were infected with increasing virus m.o.i. (Figure 2A) and cell culture supernatants were tested for the production of the proinflammatory cytokine IL-6. Cytokine production upon treatment with LPS, a TLR4 agonist, was TLR2-independent. However, at all MHV-68 m.o.i. tested, IL-6 production by TLR2-deficient MEFs was significantly reduced in comparison to WT cells. TLR2-dependent IL-6 production was detected as early as 6 hours post-infection and was sustained at 18 and 24 hours (Figure 2B). Ultraviolet (UV) irradiation of MHV-68 inhibits its potential to replicate but does not affect its structure while heat inactivation disrupts viral particle integrity [13]. Although substantially reduced in WT cells treated with UV-irradiated viruses as compared to infectious viruses, IL-6 levels still depended on TLR2 indicating that viral replication is dispensable to induce production of IL-6 upon TLR2 recognition (Figure 2C). Heat-inactivated viral preparations did not lead to IL-6 release by MEFs indicating that cytokine secretion required integrity of MHV-68 particles (data not shown). Since MyD88 is required for signaling by most TLRs, we investigated its role in IL-6 secretion in response to MHV-68. As shown in Figure 2D, IL-6 secretion induced by LPS (TLR4 agonist) and Pam_3_CSK_4_ (TLR2 agonist) was totally abolished in the absence of MyD88. The same was observed upon infection by MHV-68 highlighting the importance of the TLR2-MyD88 pathway for IL-6 secretion in response to the virus. Finally, in light of the recent findings that TLR2 activation by viral, but not bacterial ligands could lead to the production of type 1 IFN, we investigated whether TLR2-dependent MHV-68 recognition leads to type 1 IFN secretion. Lipoteichoic acid (LTA), a bacterial-derived TLR2 agonist, did not lead to IFN-α secretion by MEFs (Figure 2E). The viral synthetic agonist poly(I:C) is known to activate TLR3 via the TRIF adaptor molecule and activate IFN-α secretion. Indeed, TRIF-dependent IFN-α secretion was observed upon poly(I:C) treatment. When cells were infected with MHV-68, IFN-α production was significantly reduced in the absence of TLR2 and almost completely abolished in the absence of MyD88 while remaining unaffected in TRIF-deficient cells. MHV-68 did not lead to significant IFN-β secretion by MEFs (data not shown). Therefore, these data indicate that proinflammatory cytokine and type 1 IFN production induced by MHV-68 viral particles can occur via MyD88-dependent TLR2 signaling.

### MHV-68 activates TLR2 transcription *in vivo*

**Figure 3:**
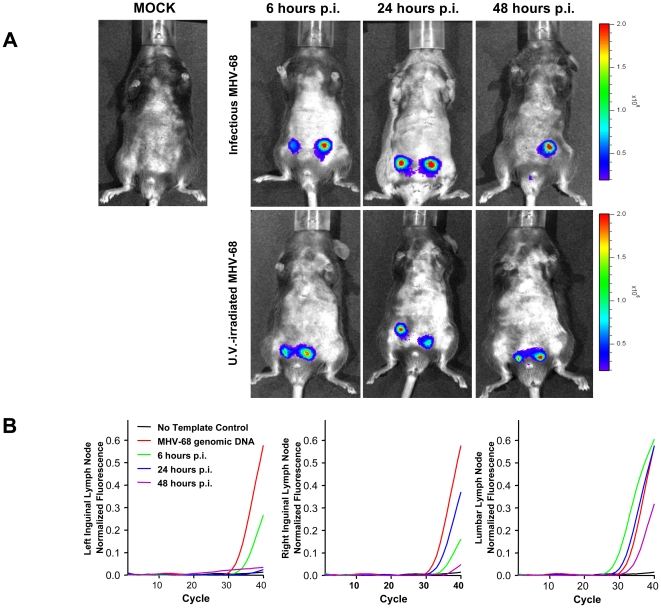
TLR2 transcription is upregulated during MHV-68 infection. (**A**) Bioluminescence assay of TLR2-LUC-AcGFP transgenic mice injected ip. with mock control, infectious MHV-68 or UV-irradiated MHV-68 for 6 hours, 24 hours and 48 hours. (**B**) Quantitative PCR of MHV-68 gB sequence from DNA extraction of inguinal and lumbar lymph nodes of mice infected with infectious MHV-68. MHV-68 genomic DNA was used as a positive control. Data are representative of three different experiments. p.i.: post-infection.

It has been shown that infection with viruses such as influenza A virus can induce an upregulation in TLR2 expression by human neutrophils [20]. This strategy may be beneficial to the virus in order to induce upregulation of viral entry receptors but may also serve the host in favoring a rapid respond to viral invaders [21], [22]. We therefore investigated whether MHV-68 could induce the activation of TLR2 transcription during an acute infection in a living animal model. For that matter, we used transgenic mice expressing a luciferase reporter gene driven under the transcriptional control of mTLR2 promoter (TLR2-luc). Since TLR2-luc was only slightly expressed in the lungs upon intranasal (in.) infection with MHV-68 (data not shown), we performed intraperitoneal (ip.) injections of the virus. As shown in Figure 3A, luciferase activity was detected in the region surrounding inguinal lymph nodes in the peritoneal cavity of TLR2-luc mice following ip. injection with MHV-68. The lack of luciferase activity in mice injected with mock control confirms the MHV-68-specific activation of TLR2 transcription. To evaluate whether viral replication is involved in the induction of TLR2 transcription, TLR2-luc mice were treated with UV-irradiated MHV-68 particles. Levels of bioluminescence observed were similar when compared to mice injected with infectious viruses indicating that the direct detection of MHV-68 particles is sufficient to induce TLR2 transcription in inguinal lymph nodes. Finally, to confirm that the bioluminescence observed was a direct consequence of MHV-68 infection, DNA from major lymph nodes present in the abdominal region (inguinal and lumbar lymph nodes) of mice was extracted and the presence of MHV-68 gB gene was tested by qPCR. As shown in Figure 3B, MHV-68 gB transcript was detected in all three lymph nodes as soon as 6 hours following infection and decreased after 48 hours of infection. Taken together, bioluminescence and qPCR data demonstrate that MHV-68 stimulation can induce transcriptional activation of TLR2 *in vivo*.

### Expression of TLR2 by cells recruited to the lungs of MHV-68 infected mice

**Figure 4:**
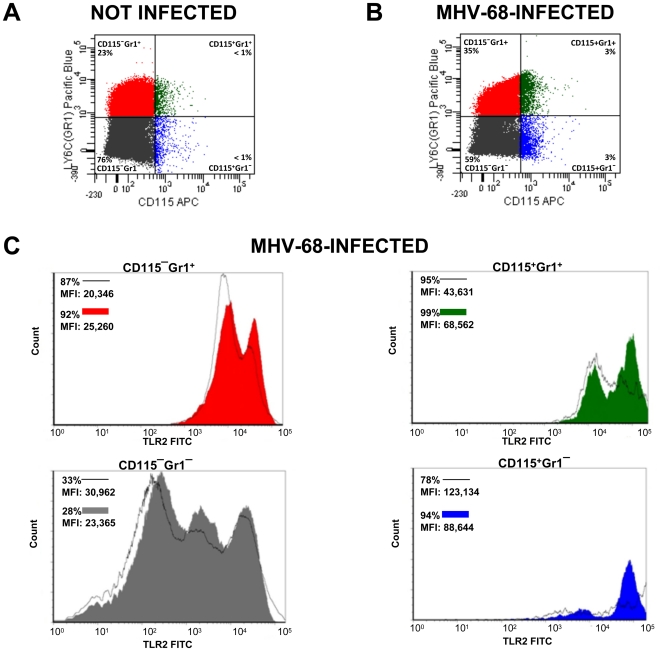
Expression of TLR2 in lungs of MHV-68-infected mice. (**A and B**) WT mice were left uninfected (**A**) or were infected in. with MHV-68 for 24 hours (**B**). Cells were isolated from lungs and stained with anti-CD115, anti-Gr1 and anti-TLR2 antibodies as detailed in the Materials and methods section. (**C**) The percentage of different cell subpopulations expressing TLR2 was determined by flow cytometry. Data are representative of two different experiments. MFI: Mean Fluorescence Intensity. Black lines: not infected. Coloured histograms: MHV-68-infected.

Monocytes and neutrophils are known to respond to microbial stimuli by trafficking to infected tissues and secreting inflammatory cytokines. Having determined that MHV-68 can activate TLR2 transcription *in vivo*, we next wanted to investigate whether lung infiltrating cells, specifically granulocytes and monocytes, express the TLR2 receptor on their surface. Mice were thus infected with MHV-68 via the in. route and the presence of those cell populations in isolated lungs was analyzed by flow cytometry. Cells were labelled with anti-CD115, anti-Ly6C (Gr1^+^), which allows to discriminate between granulocytes and monocytes (reviewed in [23]), and with anti-TLR2 antibodies to measure its expression. As shown in Figure 4, MHV-68 infection induced significant recruitment of inflammatory cells into the lungs of mice, as compared to uninfected control. Those cells seem to mainly correspond to granulocytes (CD115^−^Gr1^+^) and to two subpopulations of monocytes, one referred to as patrolling monocytes (CD115^+^Gr1^−^) and the other as inflammatory monocytes (CD115^+^Gr1^+^). This cell recruitment correlated with an increased percentage of TLR2 positive cells since granulocytes (CD115^−^Gr1^+^) and inflammatory monocytes (CD115^+^Gr1^+^) expressed higher levels of TLR2 receptor on their surface as reflected by an increased in mean fluorescence intensity (MFI). Thus, MHV-68 induces migration of TLR2-expressing inflammatory cells in the lung of infected mice.

### TLR2- and MyD88-dependent type 1 IFN production and control of viral burden following pulmonary MHV-68 infection

**Figure 5:**
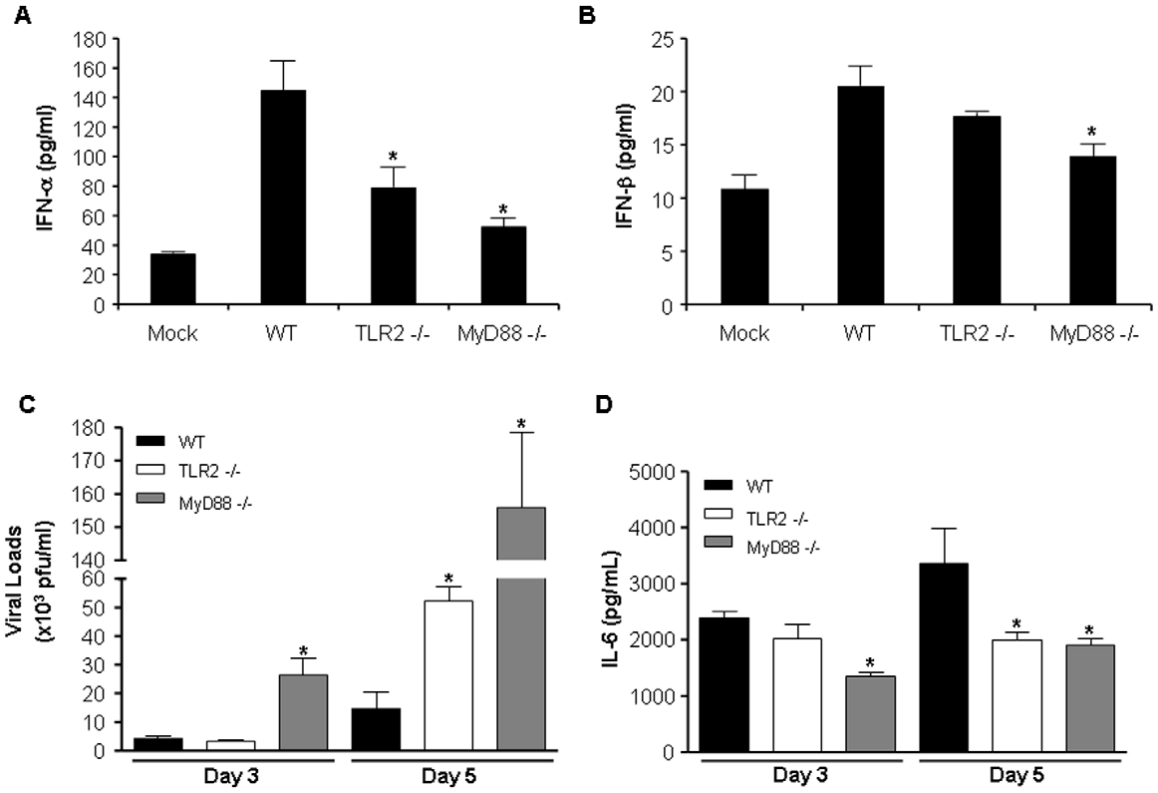
Decreased type 1 IFN and enhanced MHV-68 loads in lungs of TLR2- and MyD88-deficient mice. (**A and B**) WT, TLR2−/− and MyD88−/− mice (3 mice/group) were mock infected or infected in. with MHV-68 (10^5^ PFU/mouse). Lung homogenates were prepared 24 hours post-infection for IFN-α (**A**) and IFN-β (**B**) determination by ELISA. (**C and D**) WT, TLR2−/− and MyD88−/− mice (5 mice/group) were infected in. with MHV-68 (10^5^ PFU/mouse). Lung homogenates were prepared on days 3 and 5 post-infection for determination of viral load (**C**) and lung IL-6 concentration (**D**). Graphs are representative of three different experiments. Values significantly different (*p*<0.05) from WT mice are indicated (*).

Type 1 IFNs are critical for the control of viral infection and mice deficient in the IFN-α/β receptor are highly susceptible to MHV-68 infection ([24] and reviewed in [25]). Since we observed that TLR2 contributes to IFN-α secretion by MEFs infected with MHV-68 (Figure 2E), we wanted to determine if this was also the case *in vivo*, following pulmonary infection. WT, TLR2−/− and MyD88−/− mice were thus infected in. with MHV-68 for 24 hours and lungs were harvested for type 1 IFN determination. IFN-α levels were significantly reduced in the lungs of infected TLR2−/− and Myd88−/− mice as compared to WT mice (Figure 5A). A similar trend was observed for IFN-β albeit only reaching statistical significance for infected MyD88−/− mice (Figure 5B). In order to investigate the contribution of TLR2 in MHV-68 clearance *in vivo*, WT, TLR2- and MyD88-deficient mice were infected in. with MHV-68 and lungs were harvested at day 3 and 5 following infection for viral load determination. Increased viral load observed in TLR2−/− mice after 5 days of infection (Fig. 5C) indicates that TLR2 has a protective role in early defense against MHV-68 infection. However, the absence of increase in viral burden observed at day 3 in mice lacking TLR2 indicates that other mechanisms dependent on MyD88 signaling may be involved in viral clearance since an increase in viral titers was observed in MyD88−/− mice at both 3 and 5 days post-infection. We also assessed the level of IL-6 secretion in lungs of mice treated under the same experimental conditions. After 3 days of infection with MHV-68, both WT and TLR2−/− mice secreted similar IL-6 levels in the lung tissue, while MyD88−/− mice showed reduced IL-6 lung concentrations (Fig. 5D). At day 5 post-infection, both MyD88−/− and TLR2−/− mice had reduced lung IL-6 concentrations when compared to WT mice. Therefore, TLR2 signaling via MyD88 is important to control viral replication in the early stages of pulmonary MHV-68 infection.

### TLR2 mediates production of inflammatory mediators in different tissues

**Figure 6:**
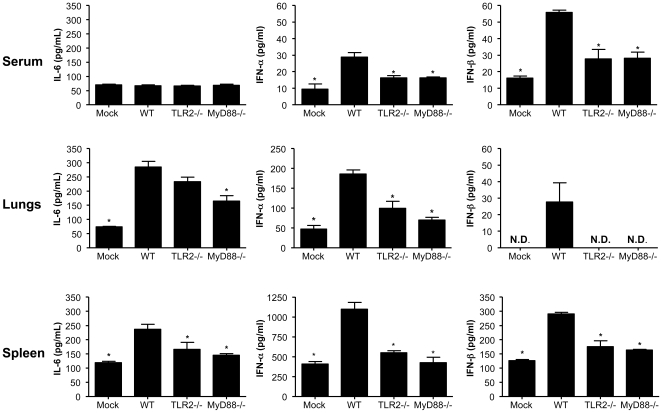
Cytokine production in sera, lungs and spleens of mice upon MHV-68 treatment. WT, TLR2−/− and MyD88−/− mice were injected iv. with mock control or UV-irradiated MHV-68 particles and sacrificed at 10 or 24 hours post-treatment. Concentration levels of IL-6 and IFN-α were determined by ELISA in sera, lung and spleen homogenates after 24 hours. IFN-β levels were tested at 10 hours post-treatment. Data are representative of two different experiments. Values significantly different (*p*<0.05) from WT mice are indicated by (*). N.D.: not detected.

To further investigate whether recognition of MHV-68 by TLR2 is restricted or not to the lungs, we compared the production of IL-6 and type 1 IFN in different tissues during systemic challenge with MHV-68. This time, WT, TLR2−/− and MyD88−/− mice were treated by intravenous (iv.) administration of UV-irradiated MHV-68 particles to avoid interference with other sensors activated by viral replication, and secretion levels of IL-6, IFN-α and IFN-β were assessed in sera, lungs and spleens. As shown in Figure 6, production of IL-6 was partially reduced in lungs and significantly reduced in spleens of TLR2-deficient mice. Such reduction was enhanced in the lungs and similar in the spleen of MyD88−/− mice suggesting that secretion of IL-6 in response to MHV-68, especially in the lungs, could be regulated through other mechanisms than TLR2 alone. On the other hand, production of IFN-α and IFN-β was similarly reduced in all tissues isolated from both strains of deficient mice as compared to WT animals. Therefore, TLR2-dependent MHV-68 recognition upon systemic challenge is not restricted to the lungs and contributes to early IL-6 and type 1 IFN secretion in multiple tissues.

## Discussion

MHV-68 is known to induce the secretion of a wide range of cytokines including IL-6, IL-10, IL-12, type 1 IFN, and IFN-γ [17], [26], [27]. In this report, we demonstrate that secretion of the proinflammatory cytokine IL-6 and of type 1 IFN induced by MHV-68 significantly relies on TLR2- and MyD88-dependent signaling. Importantly, TLR2−/− mice were impaired in their ability to control viral replication in the lungs after five days of infection, suggesting that an effective response against MHV-68 is regulated through this cell membrane receptor. TLR2 gene expression was rapidly induced following MHV-68 challenge, an event that did not require viral replication. In addition, we found that granulocytes and inflammatory monocytes migrate from the blood to the lungs of MHV-68-infected mice and express high levels of surface TLR2, suggesting that these cells may contribute to viral clearance and possibly play a relevant role as cytokine producers via TLR2 activation. However, we cannot exclude that resident cells of the respiratory tract and lungs expressing TLR2 also participate in the recognition of MHV-68. Our results also suggest that TLR2 is not the sole MyD88-dependent receptor implicated in the antiviral response against the virus since MyD88−/− mice were highly susceptible to infection. While TLR2 action appears to be central for efficient lung innate immunity regulated in part by cytokine and more importantly by type 1 IFN secretion, other MyD88-dependent mechanisms may act in synergy with TLR2 for the regulation of protective immunity against MHV-68. For example, other TLRs known to signal via MyD88 such as TLR7, TLR8, and TLR9 have been reported to play crucial roles in antiviral defense against other herpesviruses ([9], [14], [28], [29] and reviewed in [30]). The implication of these receptors in the early lung innate immune response against MHV-68 remains to be clarified.

Two different reports have investigated the implication of TLR signaling during acute MHV-68 infection. Using in. infection with a low dose of virus, Gargano *et al.* [15] observed that early viral replication in the lungs is unaffected in MyD88-deficient mice when compared to WT mice. Guggemoos *et al.* [14] investigated the implication of TLR9 signaling in the control of MHV-68. Using a high dose of virus, they observed that lung viral loads were comparable in both WT as well as TLR9−/− mice following in. infection. On the other hand, when MHV-68 infection was performed via the ip. route, spleen viral loads were more elevated in TLR9−/− mice as compared to WT mice. Our results suggest that TLR2 and downstream signaling pathways dependent on MyD88 are important assets for early MHV-68 viral control in the lungs following in. infection with a high but sublethal dose of virus. The discrepancies between these different observations might be due to several factors including the dose of virus inoculated, the infection route, as well as the innate mechanism responsible for virus clearance in a particular organ. While a low dose of virus during in. infection seems to be managed in a MyD88-independent fashion, TLR2 and MyD88 are important for proinflammatory cytokine and type 1 IFN secretion as well as for the control of pulmonary MHV-68 loads following infection with a high dose of virus. The direct implication of MyD88-dependent TLR2 signaling in MHV-68 control by lung epithelial cells, macrophages and by infiltrating neutrophils and monocytes has yet to be investigated and should clarify the role of this TLR pathway during acute MHV-68 infection. Guggemoos *et al.* observed that during ip. infection with MHV-68, TLR9 seems to play a crucial role in spleens for an efficient viral control, but not in lungs after in. infection. This observation may be explained by the differential TLR9 tissue expression. In fact, this receptor is highly expressed in the spleen while it is modestly expressed in lung tissue [31], [32]. We observed that TLR2 and MyD88-dependent signaling seems crucial for lung defense during acute MHV-68 infection and for recognition of MHV-68 particles in the lungs and spleen. These findings highlight the importance of organ-specific innate responses against MHV-68.

We have shown that MHV-68 stimulation leads to an upregulation of TLR2 transcription *in vivo*. Since viral replication is dispensable for such modulation, we can postulate that this event is the consequence of a fast activation of the innate immune system against acute MHV-68 infection. It is known that TLR2 can be internalized to the Golgi apparatus following the binding to LTA and it was also suggested that receptor internalization is required for TLR2-dependent production of type 1 IFN [18], [33]. Hypothetically, this receptor trafficking event could be one of the strategies used by the host for effective viral recognition and increased antiviral response against MHV-68. In fact, assuming that cells are able to increase detection of MHV-68 through TLR2, this would imply a better response against MHV-68 and therefore a reduction in viral burden. TLR2 upregulation is naturally absent in TLR2−/− mice and might be reduced in MyD88−/− mice, which could in part explain the reduction observed in IL-6 and type 1 IFN secretion because of a lack of a sufficient amount of functional TLR2 receptors. Therefore, the increase in viral burden in the absence of MyD88 and TLR2 proteins could be a direct result of the lack of TLR2 upregulation and associated cytokine secretion.

Type 1 IFNs are crucial toward the control MHV-68 during acute infection [24]. A recent study by Barbalat *et al.* [18] showed that TLR2 on inflammatory monocytes could recognize non-nucleic acid components of viruses and induce production of type 1 IFN. Our results showing that TLR2 is both upregulated on the surface of lung-migrating inflammatory monocytes and contributes to the production of type 1 IFN in MHV-68-infected mice are consistent with this report. However, whether inflammatory monocytes are actual type 1 IFN producers following their migration to the lungs remains to be confirmed. Furthermore, while IFN-α/β secretion was significantly inhibited in different tissues of TLR2−/− and MyD88−/− mice, residual secretion could still be detected indicating that alternative pathways, independent of MyD88, may participate and/or compensate for such secretion following MHV-68 stimulation. For example, other pathways might include viral detection systems such as RIG-I-like receptors (RLR), NOD-like receptors (NLRs) as well as TLR signaling via the TRIF adaptor protein. These hypotheses are now under investigation.

Together, the results presented in this study indicate that TLR2 is important for the activation of a rapid and efficient innate response in the lungs following acute MHV-68 infection. Having previously reported TLR2-dependent recognition of EBV by human monocytes [13] and more recently, TLR9-mediated recognition of this virus by human monocytes and plasmacytoid dendritic cells [28], it will be important to investigate whether defects in these pathways are associated with EBV pathogenesis in humans.

## Materials and Methods

### Ethics Statement

All experimental protocols using mice were performed in accordance with an internal review board-approved protocol at CHUQ Research Center (Centre Hospitalier de l'Université Laval). This study was approved by the *Comité de protection des animaux du CHUQ* (approval #09-146-1).

### Mice and MEFs culture

C57Bl/6 mice, 4–6 weeks old, were purchased from Charles River (Quebec, Canada). TLR2- and MyD88-deficient mice were kindly provided by Dr. Serge Rivest (CHUQ Research Center, Université Laval, Quebec, Canada). TRIF-deficient mice were kindly provided by Dr. T.-j. Lin (Dalhousie University, Halifax, Canada). TLR2-LUC-AcGFP transgenic mice were generated in the Transgenic and Knockout Facility of Research Centre of the Centre Hospitalier de l'Université Laval, as previously reported [34]. Murine embryonic fibroblasts (MEFs) were obtained following overnight tryptic digestion (0.5% trypsin-EDTA solution at 4°C) of 12 to 15-day-old embryos of C57Bl/6, TLR2-, MyD88- and TRIF-deficient mice as detailed [35]. MEFs were cultured in minimum essential medium (MEM) supplemented with 20% heat-inactivated fetal bovine serum (FBS).

### Viral preparation and titration

MHV-68 g2.4 strain, kindly provided by Dr. Bernadette Dutia (University of Edinburgh, Edinburgh, UK), was amplified in baby-hamster kidney (BHK-21) (ATCC, Manassas, VA, USA) cell line as described previously [36]. Cells infected with purified virus were cultured in MEM supplemented with 5% heat-inactivated FBS until 90% of mortality was observed. Supernatant was then filtered on 0.45 µm pore filter and viral particles were concentrated by ultracentrifugation [37]. Uninfected-BHK-21 cells supernatant was treated as above to produce mock control. Both viral preparations and mock control were resuspended in MEM medium for *in vitro* experiments or in saline solution (0.45% w/v NaCl containing 0.25% w/v dextrose) for *in vivo* experiments. UV-irradiated viral particles were obtained following incubation of viral preparation under UV radiation (30 min, 265 nm) [38]. Viral load determination was performed by serial dilutions using a standard plaque assay on Vero cell cultures as described [39].

### Mice infection

Intranasal (in.) infections were performed with 25 µl of MHV-68 preparation (10^5^ viral particles) in saline solution. Intraperitoneal (ip.) and intravenous (iv.) stimulations were performed using 100 µl of infectious particles (10^5^ PFU) or UV-irradiated viral preparation (10^5^ or 10^7^ viral particles). Inguinal and lumbar lymph nodes of TLR2-LUC-AcGFP mice were isolated and resuspended in TRIzol reagent (Invitrogen, Ontario, Canada) for DNA isolation. At indicated time, sera were collected by cardiac puncture and tested for IL-6 and IFN-α/β by ELISA. Lungs and spleens were also harvested and triturated for cytokine determination. Lung viral loads were determined by standard plaque assay. For flow cytometry analysis, harvested lungs were treated with collagenase to obtain single-cell suspension as described [40].

### Luciferase assay

Human embryonic kidney (HEK293) cell line (ATCC, Manassas, VA, USA) was cultured in Dulbecco modified Eagle medium (DMEM) supplemented with 10% heat-inactivated FBS. HEK293 (5×10^4^ cells/ml) were transiently co-transfected with 400 ng of either pDUO2-hMD2-CD14 (control human plasmid) or pUNO-mTLR2-HA plasmids (Invivogen, San Diego, CA, USA), using Escort transfection reagent (Sigma-Aldrich, Ontario, Canada) along with 100 ng of NF-κB luciferase reporter plasmid. Forty-eight hours following transfection, cells were stimulated for 6 hours with either mock, MHV-68 or the TLR2 ligand Pam_3_CSK_4_ (1 µg/ml). Following stimulation, cells were lysed in luciferase buffer (1% Triton, 10% glycerol, 20 mM Tris phosphate, pH 7.8) and luciferase activity was measured by luminometry. Relative light units (RLU) were normalized by protein dosage using BCA protein assay kit (Pierce Biotechnology, Rockford, IL, USA).

### *In vivo* bioluminescence

Images of TLR2-LUC-AcGFP transgenic mice were obtained using IVIS Imaging System (Xenogen, Alameda, CA, USA) fifteen to twenty-five minutes following ip. injection of D-Luciferin (150 mg/kg) (Gold Biotechnology, St. Louis, MO, USA) dissolved in saline solution. Mice were anesthetised under 2% isoflurane in 100% oxygen at a flow rate of 2 L/min and placed in a heated, light-tight imaging chamber. Images were collected using a high sensitivity CCD camera with wavelengths ranging from 300–600 nm for an exposure time of one minute. Bioluminescence was normalized and displayed in physical units of surface radiance (photons/s^−1^/cm^−2^/steradian^−1^) using Living Image 2.5 software (Xenogen) which expresses light intensity as a color gradient (from violet to red). Bioluminescence was considered significant when light emission was greater than mock-injected control.

### DNA extraction and PCR analyses

Total DNA was extracted from lymph nodes of mock- and MHV-68-infected mice using TRIzol reagent following the manufacturer's instructions (Invitrogen, Ontario, Canada) and amplified by qPCR using SYBR Green supermix (Invitrogen, Ontario, Canada). qPCR was performed using MHV-68 gB 20-mers (forward: 5′-ggcccaaattcaatttgcct-3′ and reverse 5′-ccctggacaactcctcaagc-3′) starting by 2 min at 50°C then 10 min at 95°C, followed by 40 cycles of denaturation at 95°C for 15 sec, 58°C for 1 min and followed by a temperature gradient from 60 to 99°C for 20 sec. Analysis was performed using RG-3000 from Corbett Research and Rotor Gene 6 software (Montreal Biotech Inc., Quebec, Canada).

### Cytokine quantification

MEFs (5×10^4^ cells/ml) were stimulated with lipopolysaccharide (LPS, 1 or 5 µg/ml), Pam_3_CSK_4_ (Pam, 1 µg/ml), LTA (10 µg/mL) or with either infectious or UV-irradiated MHV-68 particles or transfected with poly(I:C) (5 µg/mL) using Lipofectamine reagent (Invitrogen, Ontario, Canada) for the indicated periods of time and cell-free supernatants were harvested for IL-6 (eBiosciences, San Diego, CA, USA), IFN-α and IFN-β (PBL Biomedical Laboratories, Piscataway, NJ, USA) quantification by ELISA. Sera from uninfected and infected mice as well as organ homogenate supernatants were tested for IL-6, IFN-α and IFN-β by ELISA.

### Flow cytometry

Single-cell suspensions obtained from collagenase-treated lungs of mock- and MHV-68-infected mice were first stained with allophycocyanin-conjugated-anti-CD115 (eBioscience, San Diego, CA, USA), Pacific Blue-conjugated-anti-Ly6-G/Ly6-C(GR-1) (BioLegend, San Diego, CA, USA), followed by labelling with anti-mTLR2 monoclonal antibody (Invivogen, San Diego, CA, USA) and fluorescein-conjugated-anti-mouse antibody (Jackson ImmunoResearch Laboratories, West Grove, PA, USA) to determine expression levels of TLR2 on different cell populations. Flow cytometry analyses were performed using BD LSR II (BD Biosciences, Ontario, Canada) on 10,000 cells per sample.

### Statistical Analyses

Data were analyzed by one-tailed analysis of variance (ANOVA) followed by Newman-Keuls post-hoc test using PRISM5 software. Differences were considered significant at p<0.05.
